# A hypergraph transformer method for brain disease diagnosis

**DOI:** 10.3389/fmed.2024.1496573

**Published:** 2024-11-14

**Authors:** Xiangmin Han, Jingxi Feng, Heming Xu, Shaoyi Du, Junchang Li

**Affiliations:** ^1^School of Software, Tsinghua University, Beijing, China; ^2^The Institute of Artificial Intelligence and Robotics, College of Artificial Intelligence, Xi'an Jiaotong University, Xi'an, China; ^3^Shenzhen Clinical Research Center for Mental Disorders, Shenzhen Kangning Hospital and Shenzhen Mental Health Center, Shenzhen, China

**Keywords:** hypergraph computation, brain network, high-order correlation, brain disease diagnosis, transformer

## Abstract

**Objective:**

To address the high-order correlation modeling and fusion challenges between functional and structural brain networks.

**Method:**

This paper proposes a hypergraph transformer method for modeling high-order correlations between functional and structural brain networks. By utilizing hypergraphs, we can effectively capture the high-order correlations within brain networks. The Transformer model provides robust feature extraction and integration capabilities that are capable of handling complex multimodal brain imaging.

**Results:**

The proposed method is evaluated on the ABIDE and ADNI datasets. It outperforms all the comparison methods, including traditional and graph-based methods, in diagnosing different types of brain diseases. The experimental results demonstrate its potential and application prospects in clinical practice.

**Conclusion:**

The proposed method provides new tools and insights for brain disease diagnosis, improving accuracy and aiding in understanding complex brain network relationships, thus laying a foundation for future brain science research.

## 1 Introduction

The structural and functional connections of brain networks reflect the interaction and collaboration between different brain regions (1, 2). Structural connectivity is typically represented by the distribution of neural fiber tracts (3), while functional connectivity describes the synchronous activity of different brain regions during specific tasks or at rest state (4). Understanding the structural and functional connectivity of brain networks is crucial for comprehending both normal brain function and pathological states (5). For instance, abnormalities in structural connectivity may be associated with brain tissue damage, while disruptions in functional connectivity could indicate communication issues between neurons. Therefore, studying functional and structural brain networks is essential for uncovering the mechanisms underlying brain disease diagnosis.

Current research on brain networks primarily relies on techniques such as functional magnetic resonance imaging (fMRI) and diffusion tensor imaging (DTI). fMRI captures brain activity during specific tasks or at rest, revealing functional connectivity between different brain regions. DTI, on the other hand, tracks the diffusion paths of water molecules within neural fibers, providing information on the structural connectivity of the brain's white matter. Integrating data from fMRI and DTI offers a more comprehensive and enriched perspective for diagnosing brain diseases. For example, in Alzheimer's disease research, fMRI can reveal changes in functional connectivity, while DTI can demonstrate the degradation of white matter structure. In recent years, multimodal imaging techniques that combine fMRI and DTI have become mainstream in brain network research, further enhancing diagnostic accuracy and depth of understanding regarding brain diseases.

Artificial intelligence (AI) has achieved great success in various fields (6). For the brain network analysis task, graph and hypergraph methods (7–9) have shown great potential in brain network research. Graph methods represent brain networks as vertices and edges, allowing for the analysis of pairwise, low-order relationships. However, these methods have limitations, as they fail to effectively capture higher-order relationships within brain networks. For instance, traditional graph neural networks (GNNs) (10, 11) often underperform in handling complex high-order interactions (12, 13), neglecting the interactions among multiple vertices. Hypergraph methods (14) introduce hyperedges, which better model higher-order relationships in brain networks, but challenges remain in integrating functional and structural brain networks (15). Although hypergraphs can represent high-order relationships among multiple vertices, existing methods lack effective strategies for integrating information from different modalities, making it difficult to fully leverage the advantages of multimodal data. Thus, new methods are needed to address these issues and improve the accuracy and reliability of brain disease diagnosis.

This paper proposes a hypergraph Transformer (HGTrans) method for calculating high-order correlations between functional and structural brain networks. By utilizing hypergraphs, we can effectively model the high-order interactions within brain networks. The Transformer model provides robust feature extraction and integration capabilities, capable of handling complex multimodal data. Specifically, we use hypergraphs to represent high-order correlations in brain networks, including both functional and structural connectivity. Then, we propose the cross-attention Transformer module to extract features and integrate information from the hypergraphs, constructing a joint representation of the functional-structural brain network. This approach not only captures high-order functional and structural correlations but also effectively integrates information from different modalities, enhancing brain disease diagnosis performance. The main contributions of this paper are as follows:

- We propose a hypergraph-based method for modeling and computing the integration of functional and structural brain networks, effectively capturing high-order correlations. By using hypergraph modeling, we can accurately represent high-order interactions among multiple regions within brain networks, thereby enhancing our understanding and diagnosis performance of brain diseases.
- We introduce the Transformer model into hypergraph-based multimodal brain disease diagnosis, integrating diverse information from fMRI and DTI to improve diagnostic accuracy. The Transformer is conducted to refine the structural embeddings by incorporating high-order relationships derived from the functional network, thereby enhancing the diagnosis of brain diseases.
- We validated our method on the ABIDE and ADNI datasets, showing that our approach outperforms all the traditional and graph-based methods for different types of brain diseases, demonstrating its potential and application prospects in brain disease diagnosis.

## 2 Materials and methods

### 2.1 Datasets and preprocessing

The proposed method is evaluated on the ABIDE (16) and ADNI (17) datasets. We utilized the NYU1 and TCD sites of the ABIDE database in this work. Specifically, the NYU1 dataset contains 55 subjects, of which 33 subjects are autism spectrum disorder (ASD) patients and 22 subjects are normal controls (NCs). The TCD site contains 40 subjects, of which 20 subjects are ASD subjects and 20 subjects are NCs. The ADNI dataset is collected from multiple sites that study for improving the clinical trials for the prevention and treatment of Alzheimer's disease (AD). We used a subset of ADNI in this work, consisting of 39 AD patients, 62 MCI patients, and 61 NCs. Each subject has both rs-fMRI and DTI data in this work. The AAL (18) brain atlas was used to segment the regions of interest (ROIs) of the brain network. We preprocessed the original rs-fMRI via DPARSF,^1^ and the original DTI via PANDAS.

### 2.2 Method

#### 2.2.1 Preliminaries of hypergraph computation

The hypergraph computation framework models high-order correlations by using hyperedges, which represent complex relationships beyond pairwise connections, and performs collaborative computation on these high-order interactions. Each hyperedge can connect multiple vertices, allowing it to capture both low-order (pairwise) correlations and high-order correlations across larger vertex sets. This approach leverages these high-order interactions to optimize data usage and improve overall task performance.

Given a hypergraph H={V,E,W}, where V and E represent the vertex set and the hyperedge set, respectively, and **W** denotes the weight matrix of the hyperedges. The incidence matrix of the hypergraph is defined as a |V|×|E| matrix, with each entry defined as


(1) $$H(v,e)=\{\begin{array}{ll} w_{e}(v), & \text{if }v\in e\\ 0, & \text{if }v\notin e \end{array},$$

where *w*_*e*_(*v*)∈**W** represents the weight of vertex *v* within the hyperedge *e*.

#### 2.2.2 HGTrans framework

**Figure 1:**
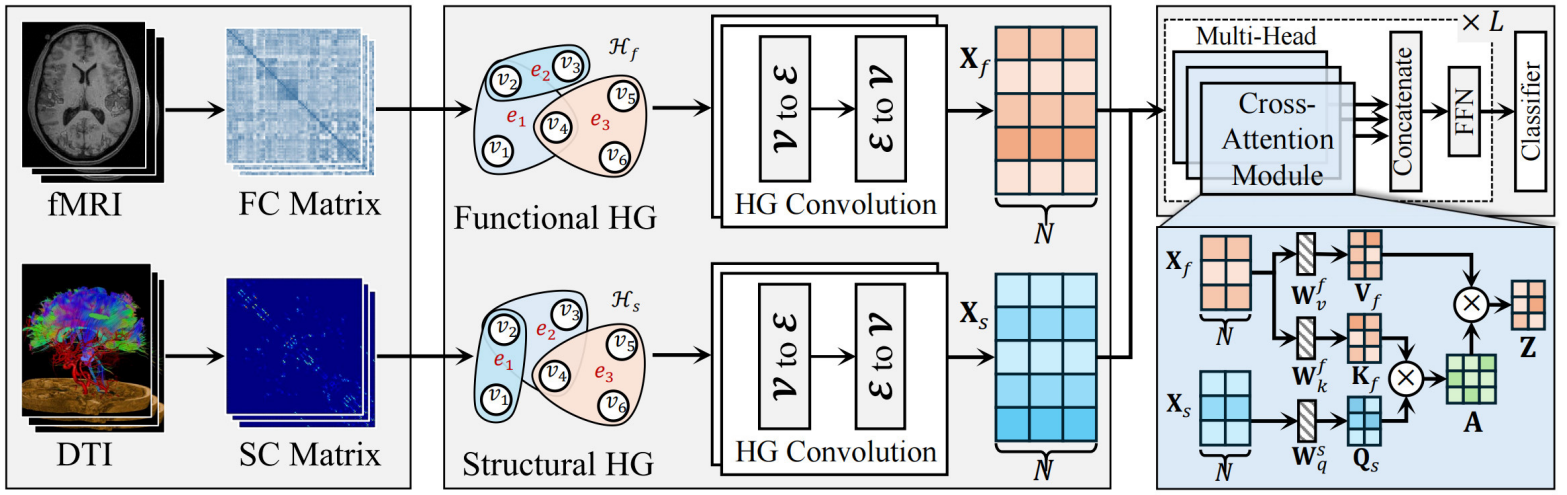
The pipeline of the proposed HGTrans framework.

As shown in Figure 1, the proposed HGTrans Framework consists of two main modules: the hypergraph computation module based on brain imaging and the structure-function Transformer module. The former constructs high-order relational structures from the information embedded in fMRI and DTI, exploring the complex relationships between different brain regions under fMRI and DTI, and generating high-order feature representations for fMRI and DTI. Then, semantic computations are performed using a hypergraph neural network to generate high-order feature representations. The latter uses the high-order features of the functional brain network as keys (K) and values (V), and the high-order features of the structural brain network as queries (Q) to achieve information interaction and fusion within the Transformer module. Finally, the fused features are fed into a classifier to enable brain disease diagnosis.

#### 2.2.3 Hypergraph computation for fMRI and DTI

##### 2.2.3.1 Higher-order functional brain network representation

To model the complex interactions within functional brain networks, we utilize hypergraphs, which allow for the connection of multiple ROIs in the brain, rather than just pairs of regions. This structure facilitates the representation of high-order associations that arise in functional brain activity. The time series data of 116 ROIs from each subject's resting-state fMRI (rs-fMRI) data is extracted, followed by calculating the Pearson correlation coefficient between each pair of ROIs. This correlation coefficient quantifies the degree of linear relationship, ranging from –1 (perfect negative correlation) to 1 (perfect positive correlation), with 0 indicating no linear association. Using this approach, a functional connectivity (FC) matrix of size 116 × 116 is then generated, where each element represents the pairwise linear correlation between two ROIs.

In the hypergraph model, each of the 116 ROIs is treated as a vertex in the set $V=\left\{v_{1},v_{2},\ldots ,v_{116}\right\}$, with *v*_*i*_ representing the *i*-th vertex. In this work, we fix the K value as 3. The vertex feature set *X*_*f*_ = {*x*_*f*1_, *x*_*f*2_, …, *x*_*f*116_} describes the Pearson correlation values between the *i*-th ROI and all other ROIs. To capture the structural relationships between ROIs, we apply a K-Nearest Neighbors (KNN) algorithm to identify the *k*_1_−1 nearest neighbors for each vertex *v*_*i*_. A hyperedge is then formed for each vertex, connecting it with its nearest neighbors. Each hyperedge *e*_*j*_, constructed using KNN with a specified *k*-value, can be expressed as *e*_*j*_ = {*v*_1_, *v*_2_, …, *v*_*k*_}, where *k* represents the number of vertices in the hyperedge. The similarity between vertices is measured using Euclidean distance, calculated as follows:


(2) $$\begin{array}{l} E_{\text{dist}}\left(v_{i},v_{j}\right)=\sqrt{\overset{d^{\left(l\right)}}{\underset{p=1}{\sum }}{\left(z_{i,p}-z_{j,p}\right)}^{2}} \end{array}$$

where *E*_dist_(*v*_*i*_, *v*_*j*_) denotes the Euclidean distance between vertices *v*_*i*_ and *v*_*j*_, and *d*^(*l*)^ represents the number of feature dimensions in layer *l*.

By incorporating KNN with multiple values of *k*, representing local and global scales, the resulting hyperedges reflect complex high-order interactions between the vertices. The functional brain network hypergraph is then used for hypergraph convolution, allowing the learning of vertex representations. The HGNN+ convolution operation (19) consists of a two-step message-passing scheme. The process is formalized as follows:


(3) $$\begin{array}{l} \;Z^{t}=WH^{T}D_{e}^{-1}X^{t}\\ X^{t+1}=\sigma (D_{v}^{-1}HZ\theta^{t+1}) \end{array}$$

where $X^{t}\in {\mathbb{R}}^{|V|\times M_{t}}$ is the vertex feature matrix at layer *t*, and $Z^{t}\in {\mathbb{R}}^{|E|\times M_{t}}$ is the corresponding hyperedge feature matrix. The learnable parameter matrix $\theta^{t+1}\in {\mathbb{R}}^{M_{t}\times M_{t+1}}$ defines the transformation for the subsequent layer. Initially, the incidence matrix *H* guides the aggregation of vertex features to generate the hyperedge feature matrix *Z*^*t*^. These features are then combined with vertex-specific hyperedge features using the learnable parameters θ^*t*^, updating the vertex feature matrix *X*^*t*+1^. A nonlinear activation function σ(·) is applied to facilitate the transformation of features.

The vertex embeddings derived from multiple layers of hypergraph convolution effectively capture high-order relationships between ROIs within the functional brain network. This modeling approach provides a superior representation of complex brain activity patterns.

##### 2.2.3.2 High-order structural brain networks

DTI data is utilized to derive the structural connectivity (SC) matrix, which quantifies the fiber tract connections between various ROIs in the brain. This method facilitates a comprehensive evaluation of potential alterations in the structural brain network that may be associated with ASD, offering a holistic perspective on how the disease may impact brain function.

The structural brain network is characterized by features such as small-world architecture and rich-club organization, both of which are critical for understanding network efficiency and communication. High-order structural characteristics are captured by computing the clustering coefficient *c*_*i*_ and degree centrality *d*_*i*_ for each ROI, based on the SC matrix. The clustering coefficient assesses the extent of local interconnectivity, while degree centrality indicates the relative importance of each region within the broader network. These metrics provide valuable insights into the efficiency of information processing and communication within and between local brain regions.

The feature representation for each vertex in the network is defined as *X*_*s*_ = {*x*_*s*1_, *x*_*s*2_, …, *x*_*s*116_}, where *x*_*si*_ represents the feature vector for the *i*-th ROI, with *x*_*si*_ = {*c*_*si*_, *d*_*si*_}. These initial features serve as input for subsequent analysis and modeling. To capture the higher-order relationships between ROIs, a K-Nearest Neighbors (KNN) algorithm is employed to construct a hypergraph representation of the structural brain network. This hypergraph captures multi-dimensional interactions that extend beyond simple pairwise connections, allowing for a more detailed representation of the complex inter-regional relationships in the brain.

Following the construction of the hypergraph, HGNN+ (19) is applied for feature learning and information integration. The hypergraph convolution process mirrors the procedure used for the functional brain network, as indicated in Equation 3. After two layers of hypergraph convolution, the resulting vertex embeddings encode high-order structural features, which are used as the final representations of each brain region. These embeddings enable a more nuanced analysis of the structural brain network, particularly in understanding the structural alterations associated with ASD. This approach provides a rigorous framework for examining both local and global connectivity patterns within the brain, offering valuable insights into the structural mechanisms underlying ASD.

##### 2.2.3.3 Cross-attention transformer for multimodal integration

To effectively fuse functional and structural brain network features, a cross-attention Transformer module is introduced. This module leverages the Transformer architecture to model long-range dependencies between multimodal features, using the structural embeddings after hypergraph convolution as the query (Q) and the functional embeddings as the key (K) and value (V), enabling the integration of both modalities.

First, the structural embedding matrix *X*_*s*_ and the functional embedding matrix *X*_*f*_ obtained from hypergraph convolution are projected into Q, K, and V representations as follows:


(4) $$\begin{array}{l} Q_{s}=W_{q}^{s}X_{s},\;K_{f}=W_{k}^{f}X_{f},\;V_{f}=W_{v}^{f}X_{f}, \end{array}$$

where $W_{q}^{s}$, $W_{k}^{f}$, and $W_{v}^{f}$ are learnable weight matrices that linearly project the structural and functional embeddings. This step maps both sets of features into a shared feature space, preparing them for cross-attention.

Next, through the cross-attention mechanism, the query matrix *Q*_*s*_ from the structural features attends to the key matrix *K*_*f*_ from the functional features, generating the attention weight matrix:


(5) $$\begin{array}{l} A=\text{softmax}\left(\frac{Q_{s}K_{f}^{T}}{\sqrt{d_{k}}}\right), \end{array}$$

where *A* represents the attention weight matrix, and *d*_*k*_ is the dimensionality of the key, used for scaling. These attention weights are then applied to the value matrix *V*_*f*_ from the functional features to generate updated structural feature embeddings:


(6) $$\begin{array}{l} Z=AV_{f}. \end{array}$$

This process refines the structural embeddings by incorporating high-order relationships derived from the functional network, enabling a more comprehensive representation of brain activity.

##### 2.2.3.4 Brain disease diagnosis

The learned feature representations from the cross-attention Transformer module are then fed into the output layer for classification. The output layer consists of a fully connected layer and a log_softmax activation function to facilitate the final classification prediction.

Let *x*_*i*_ and *b*_*i*_ represent the input and bias for the *i*-th hidden layer, respectively, while *W*_*i*_ denotes the weight matrix facilitating connections from the *i*-th to the *i*+1-th hidden layer. Subsequently, the activation of the *i*+1-th hidden layer is computed using the equation below:


(7) $$\begin{array}{l} Z_{i+1}=f\left(W_{i}x_{i}+b_{i}\right), \end{array}$$

*Z*_*i*+1_ is the activated output of layer *i*+1.*f* denotes the activation function.


(8) $$\begin{array}{l} f\left(x\right)=\max\left(0,x\right), \end{array}$$

The ReLU activation function constrains its output to the range [0, ∞). For the final layer, a fully connected dense layer is paired with a log_softmax activation functionactivation function to execute the terminal classification predictions.


(9) $$\begin{array}{l} \text{log-softmax}\left(z_{i}\right)=z_{i}-\log\left(\underset{j}{\sum }e^{z_{j}}\right), \end{array}$$

The log-softmax function, which uses Euler's number *e* as the base for the natural logarithm. In a binary classification setting, it provides log probabilities as the output. We utilize the Adam optimizer for the optimization process, setting a relatively low learning rate of 1 × 10^−5^. The negative log-likelihood loss function is utilized for the binary classification task, the loss function is defined as follows:


(10) $$\begin{array}{l} L=-\log\left(p_{y}\right) \end{array}$$

where *p*_*y*_ represents the probability of the correct class *y*.

## 3 Results and discussion

The proposed method is compared against four categories of methods:

- Single-modality-based baseline: SVM (20), MLP (21)
- Single-modality-based graph methods: GCN (10), GAT (11), and GraphSage (22).
- Single-modality-based hypergraph method: HGNN+ (19).
- Multi-modality-based methods: BrainNN (23) and MVGCN (24).

A three-fold cross-validation approach was utilized to evaluate each method, quantifying the accuracy of ASD disease classification predictions using metrics such as accuracy, sensitivity, specificity, and F1 score. The final results is given by mean ± standard error. Tables 1, 2 show the experimental results of ABIDE and ADNI, respectively.

**Table 1:** The comparison results on the two ABIDE datasets.

**Modality**	**Method**		**Accuracy**	**Sensitivity**	**Specificity**	**F1-score**
**ABIDE-NYU**						
fMRI	SVM	(mean ± std)	0.746 ± 0.024	0.879 ± 0.043	0.547 ± 0.034	0.633 ± 0.024
	MLP	(mean ± std)	0.655 ± 0.048	0.879 ± 0.086	0.327 ± 0.185	0.400 ± 0.163
	GCN	(mean ± std)	0.618 ± 0.046	0.909 ± 0.074	0.185 ± 0.072	0.274 ± 0.090
	GAT	(mean ± std)	0.617 ± 0.053	0.667 ± 0.086	0.542 ± 0.083	0.590 ± 0.084
	GraphSage	(mean ± std)	0.582 ± 0.068	0.606 ± 0.086	0.542 ± 0.083	0.509 ± 0.077
	HGNN+	(mean ± std)	0.707 ± 0.098	0.849 ± 0.113	0.494 ± 0.149	0.672 ± 0.117
	BrainGB	(mean ± std)	0.691 ± 0.068	0.849 ± 0.043	0.452 ± 0.121	0.651 ± 0.085
	BrainGNN	(mean ± std)	0.727 ± 0.046	0.818 ± 0.074	0.583 ± 0.131	0.703 ± 0.056
DTI	SVM	(mean ± std)	0.582 ± 0.068	0.818 ± 0.074	0.185 ± 0.072	0.509 ± 0.077
	MLP	(mean ± std)	0.563 ± 0.011	0.818 ± 0.074	0.232 ± 0.139	0.244 ± 0.063
	GCN	(mean ± std)	0.527 ± 0.068	0.788 ± 0.086	0.143 ± 0.202	0.154 ± 0.218
	GAT	(mean ± std)	0.511 ± 0.078	0.697 ± 0.043	0.232 ± 0.139	0.266 ± 0.139
	GraphSage	(mean ± std)	0.545 ± 0.033	0.758 ± 0.113	0.232 ± 0.139	0.270 ± 0.112
	HGNN+	(mean ± std)	0.637 ± 0.0 61	0.758 ± 0.113	0.548 ± 0.034	0.622 ± 0.056
fMRI&DTI	BrainNN	(mean ± std)	0.688 ± 0.142	0.788 ± 0.086	0.536 ± 0.278	0.669 ± 0.049
	MVGCN	(mean ± std)	0.748 ± 0.104	0.909 ± 0.091	0.512 ± 0.238	0.602 ± 0.206
	HGTrans	(mean ± std)	0.799 ± 0.0957	0.909 ± 0.074	0.631 ± 0.144	0.778 ± 0.110
**ABIDE-TCD**				
fMRI	SVM	(mean ± std)	0.625 ± 0.112	0.650 ± 0.200	0.600 ± 0.255	0.628 ± 0.101
	MLP	(mean ± std)	0.646 ± 0.136	0.500 ± 0.175	0.794 ± 0.900	0.636 ± 0.140
	GCN	(mean ± std)	0.625 ± 0.126	0.794 ± 0.147	0.468 ± 0.258	0.526 ± 0.018
	GAT	(mean ± std)	0.623 ± 0.072	0.667 ± 0.294	0.611 ± 0.235	0.595 ± 0.112
	GraphSage	(mean ± std)	0.623 ± 0.072	0.556 ± 0.192	0.706 ± 0.107	0.652 ± 0.045
	HGNN+	(mean ± std)	0.676 ± 0.067	0.659 ± 0.124	0.363 ± 0.059	0.500 ± 0.082
	BrainGB	(mean ± std)	0.691 ± 0.068	0.849 ± 0.043	0.452 ± 0.121	0.651 ± 0.085
	BrainGNN	(mean ± std)	0.698 ± 0.070	0.746 ± 0.081	0.651 ± 0.059	0.697 ± 0.069
DTI	SVM	(mean ± std)	0.582 ± 0.068	0.818 ± 0.074	0.185 ± 0.072	0.509 ± 0.077
	MLP	(mean ± std)	0.526 ± 0.065	0.508 ± 0.259	0.548 ± 0.236	0.514 ± 0.112
	GCN	(mean ± std)	0.527 ± 0.068	0.788 ± 0.086	0.143 ± 0.202	0.154 ± 0.218
	GAT	(mean ± std)	0.601 ± 0.127	0.651 ± 0.059	0.564 ± 0.224	0.568 ± 0.166
	GraphSage	(mean ± std)	0.500 ± 0.031	0.540 ± 0.157	0.437 ± 0.224	0.436 ± 0.165
	HGNN+	(mean ± std)	0.498 ± 0.136	0.349 ± 0.059	0.667 ± 0.294	0.482 ± 0.123
fMRI&DTI	BrainNN	(mean ± std)	0.672 ± 0.080	0.444 ± 0.098	0.897 ± 0.074	0.732 ± 0.067
	MVGCN	(mean ± std)	0.698 ± 0.113	0.659 ± 0.170	0.746 ± 0.081	0.714 ± 0.101
	HGTrans	(mean ± std)	0.749 ± 0.098	0.970 ± 0.043	0.698 ± 0.022	0.748 ± 0.097

**Table 2:** The comparison results on the ADNI dataset.

**Modality**	**Method**		**Accuracy**	**Sensitivity**	**Specificity**	**F1-score**
fMRI	SVM	(mean ± std)	0.709 ± 0.041	0.487 ± 0.096	0.852 ± 0.001	0.563 ± 0.079
	MLP	(mean ± std)	0.700 ± 0.050	0.410 ± 0.131	0.886 ± 0.002	0.644 ± 0.077
	GCN	(mean ± std)	0.639 ± 0.068	0.462 ± 0.109	0.721 ± 0.050	0.591 ± 0.082
	GAT	(mean ± std)	0.660 ± 0.026	0.436 ± 0.096	0.805 ± 0.064	0.618 ± 0.037
	HGNN+	(mean ± std)	0.710 ± 0.037	0.641 ± 0.192	0.756 ± 0.099	0.689 ± 0.054
	BrainGB	(mean ± std)	0.690 ± 0.063	0.513 ± 0.254	0.802 ± 0.073	0.646 ± 0.097
	BrainGNN	(mean ± std)	0.700 ± 0.004	0.487 ± 0.036	0.837 ± 0.019	0.665 ± 0.009
DTI	SVM	(mean ± std)	0.650 ± 0.023	0.539 ± 0.126	0.724 ± 0.115	0.539 ± 0.045
	MLP	(mean ± std)	0.690 ± 0.059	0.436 ± 0.158	0.853 ± 0.037	0.640 ± 0.086
	GCN	(mean ± std)	0.683 ± 0.085	0.571 ± 0.106	0.756 ± 0.099	0.664 ± 0.089
	GAT	(mean ± std)	0.669 ± 0.068	0.436 ± 0.096	0.818 ± 0.065	0.628 ± 0.075
	HGNN+	(mean ± std)	0.690 ± 0.039	0.462 ± 0.109	0.837 ± 0.019	0.649 ± 0.057
fMRI&DTI	BrainNN	(mean ± std)	0.701 ± 0.045	0.487 ± 0.131	0.838 ± 0.097	0.661 ± 0.055
	MVGCN	(mean ± std)	0.690 ± 0.050	0.462 ± 0.063	0.837 ± 0.081	0.538 ± 0.061
	HGTrans	(mean ± std)	0.740 ± 0.050	0.539 ± 0.109	0.851 ± 0.109	0.698 ± 0.049

### 3.1 Comparison with single-modality baseline methods

In Tables 1, 2, single-modality baseline methods include traditional machine learning approaches such as Support Vector Machines (SVM) and Multilayer Perceptron (MLP). While these methods are widely used for classification tasks, they are limited to features from a single modality and cannot capture the complex interactions within brain networks. Specifically, on the ABIDE-NYU dataset, SVM achieved an accuracy of 0.746, and MLP achieved 0.655, both lower than the accuracy of 0.799 achieved by our proposed HGTrans. Similarly, on the ABIDE-TCD dataset, SVM and MLP achieved 0.625 and 0.646, respectively, which are significantly lower than HGTrans's 0.749. These results indicate that single-modality baseline methods are insufficient for effectively addressing the complexity of ASD data. By integrating both fMRI and DTI data, HGTrans can capture more informative features from different perspectives of the brain network, leading to superior classification performance. This highlights the necessity and effectiveness of multimodal data fusion.

### 3.2 Comparison with graph-based methods

Graph-based methods, including GCN, GAT, and GraphSAGE, utilize the graph structure of brain networks to model relationships between regions of interest (ROIs). These methods can capture more complex spatial topological features than traditional single-modality methods. However, as shown in Table 1, on the ABIDE-NYU dataset, GCN achieved an accuracy of 0.618, GAT 0.617, and GraphSAGE 0.582, all significantly lower than HGTrans's 0.799. Similarly, on the ABIDE-TCD dataset, GCN, GAT, and GraphSAGE achieved accuracies of 0.625, 0.623, and 0.623, respectively, which are lower than HGTrans's 0.749. Although graph-based methods can capture the topological information within brain networks, they are limited to modeling pairwise relationships and cannot fully represent higher-order interactions between brain regions. In contrast, HGTrans leverages hypergraph modeling to capture more complex higher-order relationships in multimodal settings, which significantly improves classification performance over traditional graph methods.

### 3.3 Comparison with hypergraph-based methods

Hypergraph-based methods, such as HGNN+, extend the capabilities of graph models by capturing higher-order relationships between multiple brain regions through hypergraph structures. On the ABIDE-NYU dataset, HGNN+ achieved an accuracy of 0.707, and on the ABIDE-TCD dataset, it achieved 0.676, both close to but lower than HGTrans's 0.799 and 0.749, respectively. These results show that while hypergraph methods can capture more complex brain region interactions, performance remains limited when using single-modality data. HGTrans outperforms HGNN+ primarily due to its ability to not only capture higher-order spatial topological structures through hypergraphs but also effectively integrate functional and structural brain network features using cross-attention mechanisms. By jointly modeling multimodal data, HGTrans generates more robust embeddings, leading to superior performance compared to single-modality hypergraph methods. On the other hand, When we engage in cognitive activities such as reading, writing, and listening, multiple brain regions cooperate to complete the tasks (25, 26), rather than a single brain region or pairs of brain regions working independently. Traditional methods find it difficult to model such group high-order correlations. However, high-order correlation modeling and semantic computation based on hypergraphs can achieve high-order correlation-driven local brain region cooperative message passing, which is more efficient than traditional graph neural networks and contains richer information. Therefore, for brain disease diagnosis tasks, the hypergraph computation model can provide more abundant semantic information, thereby improving diagnostic performance.

### 3.4 Comparison with multimodal methods

Multimodal methods, such as MVGNN and BrainNN, integrate both fMRI and DTI data to capture complementary information from different brain modalities. As shown in Tables 1, 2, while these multimodal methods outperform single-modality and graph-based methods, HGTrans still achieves the highest accuracy across both datasets. On the ABIDE-NYU dataset, MVGNN achieved an accuracy of 0.748, and BrainNN 0.688, both lower than HGTrans's 0.799. On the ABIDE-TCD dataset, MVGNN, and BrainNN achieved accuracies of 0.698 and 0.672, respectively, also lower than HGTrans's 0.749. HGTrans's advantage lies in its ability to not only fuse multimodal data but also effectively capture the complex interactions between functional and structural brain networks through hypergraph structures and cross-attention mechanisms. This mechanism allows the model to fully leverage the relationships between functional and structural brain networks, resulting in more expressive features and higher classification accuracy. There are also some domain adaption methods (27, 28) that can be used to transfer knowledge between structural and functional brain imaging. Although these cross-modal information transfer methods can achieve inference with only one modality in the testing phase, the performance is greatly limited by the lack of shared labels to guide the cross modality fusion.

## 4 Conclusion

In this study, we proposed a hypergraph Transformer-based approach to model and compute high-order associations between functional and structural brain networks. Our method effectively integrates multimodal data from fMRI and DTI, overcoming the limitations of traditional graph methods that can only capture pairwise relationships. By leveraging hypergraphs to model complex higher-order interactions and employing the Transformer architecture for feature extraction and integration, our approach has demonstrated significant improvements in brain disease diagnosis. The experimental results on the ABIDE and ADNI datasets show that the proposed method consistently outperforms existing approaches, confirming its effectiveness in enhancing the accuracy of brain disease classification. The introduction of a hypergraph-based model and the application of Transformer networks provide a robust framework for multimodal brain network analysis, advancing our understanding of the relationship between structural and functional connectivity.

## Data Availability

The original contributions presented in the study are included in the article/supplementary material, further inquiries can be directed to the corresponding author.

## Footnotes

^1^ http://rfmri.org/DPARSF
